# Assessment of postoperative pain after unilateral mastectomy using two different surgical techniques in dogs

**DOI:** 10.1186/1751-0147-55-60

**Published:** 2013-08-19

**Authors:** Bruno W Minto, Lisiane C Rodrigues, Paulo VM Steagall, Eduardo R Monteiro, Claudia VS Brandão

**Affiliations:** 1 Department of Veterinary Clinics and Surgery, School of Veterinary Medicine and Animal Science, Sao Paulo State University, UNESP, Via de Acesso Prof. Paulo Roberto Castellane s/n, Jaboticabal, SP, Brazil

**Keywords:** Dog, Cancer, Mastectomy, Analgesia, Surgery

## Abstract

**Background:**

There are few studies reporting pain and postoperative analgesia associated with mastectomy in dogs. The aim of this study was to evaluate postoperative pain after unilateral mastectomy using two different surgical techniques in the dog.

**Findings:**

Twenty female dogs were assigned (n=10/group) to undergo unilateral mastectomy using either the combination of sharp and blunt dissection (SBD) or the modified SBD (mSBD) technique, in which the mammary chain is separated from the abdominal wall entirely by blunt (hand and finger) dissection except for a small area cranial to the first gland, in a prospective, randomized, clinical trial. All dogs were premedicated with intramuscular acepromazine (0.05 mg/kg) and morphine (0.3 mg/kg). Anesthesia was induced with intravenous ketamine (5 mg/kg) and diazepam (0.25 mg/kg), and maintained with isoflurane. Subcutaneous meloxicam (0.2 mg/kg) was administered before surgery. Postoperative pain was evaluated according to the University of Melbourne pain scale (UMPS) by an observer who was blinded to the surgical technique.. Rescue analgesia was provided by the administration of intramuscular morphine (0.5 mg/kg) if pain scores were >14 according to the UMPS. Data were analyzed using t-tests and ANOVA (*P*>0.05). There were no significant differences between the groups for age, weight, extubation time, and duration of surgery and anesthesia (*P*>0.05). There were no significant differences for postoperative pain scores between groups. Rescue analgesia was required in one dog in each group.

**Conclusions:**

The two surgical techniques produced similar surgical times, incidence of perioperative complications and postoperative pain. Multimodal analgesia is recommended for treatment of postoperative pain in dogs undergoing unilateral mastectomy.

## Findings

The mammary glands are frequent locations for the development of tumors in dogs [1-3]. Surgery remains the gold-standard treatment for most types of these tumors except for inoperable highly metastatic disease and for most of the inflammatory mammary carcinomas [1,2]. Unilateral mastectomy may induce an inflammatory response that leads to hyperalgesia of the surgical incision with peripheral sensitization [4]. Few studies have reported signs of postoperative pain associated with mastectomy in the dog [4-7]; clinical experience shows that aggressive pain management is usually required to provide analgesia in these patients. Furthermore, the analgesic technique may improve postoperative feeding behavior [5]. The combination of sharp and blunt dissection (SBD) is a popular surgical approach described for mastectomy in this species [1]. Alternatively, a modification of this technique (mSBD) involves using only finger dissection to separate the mammary chain from the underlying musculature. This study aimed to compare the SBD and the mSBD technique regarding their influence on postoperative pain in dogs undergoing unilateral mastectomy.

The study protocol was approved by the Animal Care and Use Committee of the São Paulo State University - UNESP Botucatu, SP, Brazil. Twenty client-owned female dogs scheduled for unilateral mastectomy because of mammary tumor were included in a prospective trial after owner’s written consent was obtained. Mammary neoplasia was diagnosed by cytological examination of fine-needle aspirates. Inclusion criteria included normal thoracic radiographs, and CBC and serum biochemical analyses within reference range and that no evidence of metastasis was detected. Any dog presenting with systemic disease, cardiac arrhythmias, pregnancy, extreme aggression or that was geriatric, obese or debilitated was excluded from the study. Unilateral mastectomy was indicated based on clinical findings and cytology of the mammary gland [8].

Dogs were premedicated with acepromazine (0.05 mg/kg; Acepram, Univet, Brazil) and morphine sulphate (0.3 mg/kg; Dimorf, Cristália) administered intramuscularly. A 20-gauge catheter was aseptically introduced into a cephalic vein and anesthesia was induced with intravenous administration of diazepam (0.25 mg/kg; Diazepam, Hipolabor) and ketamine (5 mg/kg; Ketalar, Pfizer). After endotracheal intubation, anesthesia was maintained with isoflurane in 100% oxygen. All dogs received subcutaneous meloxicam (0.2 mg/kg; Metacam, Boehringer Ingelheim) preoperatively and lactated Ringer’s solution was administered (10 ml/kg/h) throughout the procedure. A single anesthetist (PVMS) adjusted vaporizer settings to maintain surgical depth of anesthesia. Surgical procedures were performed by the same experienced surgeon (BWM). Dogs were randomly assigned to undergo unilateral mastectomy (n=10/group) using either the SBD or the mSBD technique. In the SBD group, surgery was performed as previously described [5]. Briefly, the distance between the nipple and the midline was measured. The same distance lateral to the nipple was estimated as the lateral border of the mammary glands and these landmarks were used to calculate wide margins (3 cm lateral margins and one fascial plan underneath the tumor). Then, two curved incisions of the skin and subcutaneous tissues were performed from the cranial aspect of the first to the caudal aspect of the fifth mammary gland. From cranial to the caudal aspect, the skin and subcutaneous tissues were separated along the cranial and lateral borders, and elevated from the pectoral muscles and the external abdominal fascial sheath by sharp and blunt dissection using scissors. The branches of the internal thoracic, lateral thoracic, and intercostal vessels and the cranial and caudal superficial epigastric artery and vein were ligated and the entire mammary chain was removed. The wound edges were undermined and the defect was closed in three layers. The deep and superficial subcutaneous tissues were closed with interrupted sutures and walking sutures of 2–0 PDS (polydioxanone). The skin was closed routinely with interrupted sutures of 3–0 polypropylene. The sutures were removed 2 to 3 weeks after placement. In the mSBD group, two curved incisions of the skin and subcutaneous tissues were performed from the cranial aspect of the first to the caudal aspect of the fifth mammary gland with wide margins. The skin and subcutaneous tissues were separated from the abdominal wall by sharp and blunt dissection with scissors, only cranially and laterally to the first mammary gland. The mammary tissue was firmly elevated between the glands and the external abdominal fascia and it was pulled from cranial to caudal direction with one hand. The other hand was used to dissect between the mammary glands and the pectoral muscles and the external abdominal fascial sheath along the mammary chain, and without the use of scissors. The major vessels were identified and properly ligated as described in the SBD group. It was ensured that no mammary tissue was left in place, and the mammary chain was completely removed. Sterile gloves were changed prior to wound closure in both groups. The wound edges were undermined and the defect was closed in three layers as described in the SBD group. The axillary and inguinal lymph nodes were removed in all dogs. Duration of anesthesia and surgery were recorded for each dog.

Postoperative pain was assessed using the University of Melbourne pain scale (UMPS) [9] at 1, 2, 3, 4, 6, 8, 12, and 24 hours after the end of the surgery. The scale included multiple descriptors in six categories of data or behaviors associated with response to pain. Categories included physiological data, response to palpation, activity, mental status, posture, and vocalization. A single trained observer (LCR) who was unaware of the surgery technique was present for the entire pain assessment period Rescue analgesia (0.5 mg/kg of morphine, IM) was administered if pain scores were ≥ 14 (maximum pain score of 27). At hospital discharge, postoperative analgesia included the administration of meloxicam and/or tramadol.

A Shapiro-Wilk test was used to analyze data and normality. Data are reported as mean ± standard deviation (SD) values or median (lower-upper range) where appropriate. Weight and age of dogs as well as duration of anesthesia and surgery were compared between groups by paired t tests. A Mann Whitney test was used to compare pain scores between groups at each time point and a Friedman test was used to compare differences over time within each group. Differences were considered significant at *P* <0.05.

Breeds included in this study were crossbred (n = 9), German Shepherd (3), Cocker Spaniel (2), Boxer (2), Rottweiler (2), Poodle (1) and Labrador Retriever (1). There were no significant differences between groups for demographic data. Mean ± SD of weight, age, duration of anesthesia and surgery were: 23.3 ± 9.1 and 23.7 ± 11.2 kg; 8.1 ± 3.4 and 8.5 ± 1.7 years; 66.3 ± 16.9 and 75.0 ± 13.0 minutes; 59.5 ± 15.4 and 63.9 ± 14.1 minutes (SBD and mSBD groups, respectively). There were no anesthetic or intra- and post-operative complications throughout the study period with the exception of mild inflammation of the skin incision. None of the animals were painful before surgery. However, pain scoring was not performed at baseline since some dogs were panting, anxious and/or fearful during physical examination. This could have led to a false increase in scores for physiological data, and produce differences between groups that were not related to pain per se. Pain scores were not significantly different over time (when compared to 1 h) or between groups (Table 1). Rescue analgesia was required in one dog in the SBD group at extubation and in one dog in the mSBD group at one hour. Data obtained from dogs after administration of rescue analgesia were excluded from statistical analysis.

**Table 1:** Postoperative pain scores (median and lower – upper range) in dogs undergoing unilateral mastectomy using the University of Melbourne pain scale (UMPS)

	**Group**		
**Hours**	**SBD**	**Msbd**	*P *value^*^
1	5 (2–11)	5.5 (2–8)	0.48
2	6 (4–8)	5 (2–9)	0.53
3	5 (2–10)	6 (2–8)	0.44
4	5 (0–7)	6 (3–7)	0.30
6	5 (0–8)	6 (3–7)	0.85
8	5 (1–7)	6 (4–11)	0.23
12	5 (4–7)	5 (3–6)	0.88
24	6 (4–8)	6 (1–8)	0.61
*P* value^†^	0.13	0.42	

*Comparisons between groups (Mann Whitney test); † Comparisons over time within each group (Friedman test).

*SBD* sharp and blunt dissection surgery technique, *mSBD* modified SBD.

Our results indicated that the duration of surgery, incidence of perioperative complications and postoperative pain was similar between the two studied surgical techniques. The mSBD technique was performed uneventfully in all individuals. However, in a few dogs in the mSBD group, it was difficult to separate the subcutaneous tissue from the underlying muscles. This could be due to increased adhesion of mammary tissue to the pectoral muscle [8]. The authors observed less bleeding in the mSBD group, subjectively. This was likely to be a result of the laceration and collapse of local blood supply, and further hemostasis. Despite of this subjective observation, the modified technique did not demonstrate any other advantages over the SBD technique. This study design could not rule out the recurrence of mammary tumor since no long-term follow-up was performed after surgery.

There are only few reports investigating postoperative pain in dogs undergoing mastectomy [4-7]. Unilateral mastectomy may lead to moderate pain with an obvious inflammatory component [4]. For this reason, all dogs were given morphine and meloxicam before surgery. In this study, there was no difference between the two surgical techniques in the degree of postoperative pain experienced suring the first 24 hours after surgery. In the present study dogs were administered rescue analgesia if pain scores were ≥ 14, as indicative of moderate pain. This was an arbitrary cut-off point for provision of rescue analgesia, and there is no consensus on what score should have been chosen for such intervention. However, it is clear that a lower cut-off would have been more appropriate for mild pain, and in this case the incidence of intervention analgesia could have been higher.

## Conclusions

In summary, both surgical techniques produced similar surgical times, incidence of perioperative complications and postoperative pain. Multimodal analgesia is recommended for the control of postoperative pain in dogs undergoing mastectomy.

## Abbreviations

mSBD: Modified SBD technique; SBD: Sharp and blunt dissection; SD: Standard deviation; UMPS: University of Melbourne pain scale.

## Competing interests

The authors declare that they have no competing interests.

## Authors’ contributions

BWM was the surgeon of the study. He was responsible for patient recruitment, perioperative care, study design, and manuscript drafting. Major conceiver of the study. LCR scored postoperative pain during 24 hours. Responsible for recruiting patients and for rechecks, owner’s consent forms, surgery scheduling, paperwork, including data handling. PVMS was the anesthesiologist of the study who was responsible for administering rescue analgesia and postoperative care. Participation in the study design, data analysis, and main manuscript drafting. ERM participated in the design of the study and performed the statistical analysis. Involvement with manuscript drafting. CVSB contributed to the conception and design of the study, and recruitment of patients. She was also the conceiver and coordinator of the study. All authors read and approved the final manuscript.
